# Supplementary material for: Preserving a robust CsPbI3 perovskite phase via pressure-directed octahedral tilt
Source: Nat Commun. 2021 Jan 19;12:461. doi: 10.1038/s41467-020-20745-5 (PMC7815753; doi:10.1038/s41467-020-20745-5)
Supplement: Supplementary file 1 — Supplementary Information [file 41467_2020_20745_MOESM1_ESM.pdf]

Supplementary Information for

Preserving a robust $CsPbI_3$ perovskite phase via pressure-directed octahedral tilt

Feng Ke[#], Chenxu Wang[#], Chunjing Jia[#], Nathan R. Wolf, Jiejuan Yan, Shanyuan Niu, Thomas P. Devereaux, Hemamala I. Karunadasa, Wendy L. Mao, Yu Lin*

[#] F.K., C.W., and C.J. contributed equally to this work

*To whom correspondence should be addressed. E-mail: lyforest@stanford.edu

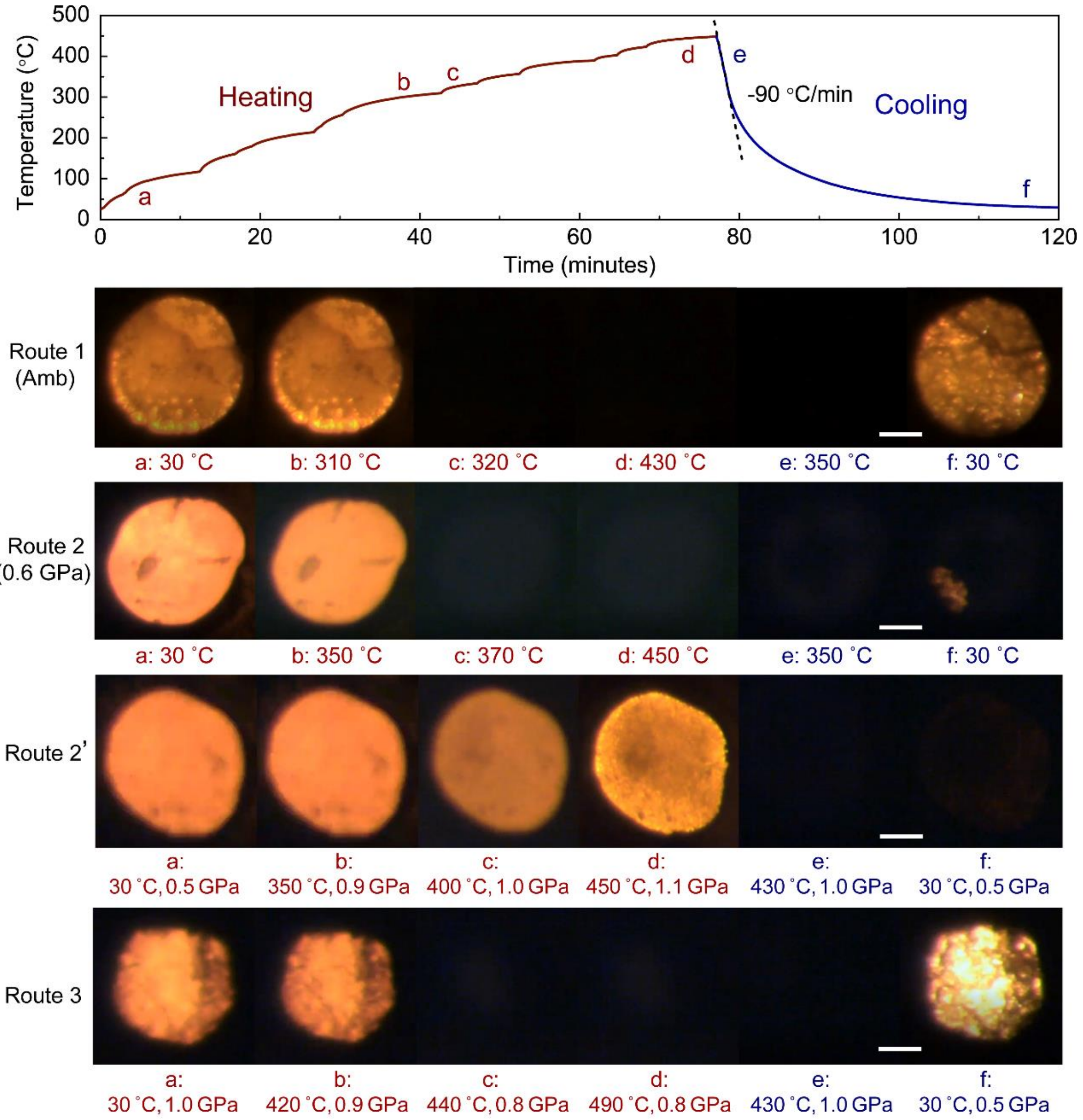

Supplementary Figure 1. A representative temperature profile for heating and rapid cooling cycles (top panel) and optical images (bottom four panels) of $CsPbI_3$ in a diamond anvil cell during varying *P*-*T* routes shown in Fig. 3a. The temperature profiles were similar except for the difference in the highest heating temperature. All of the rapid cooling routes had a similar cooling rate of -90 ºC/min. A fan was used to cool down the samples after turning off the power of the resistive heater. The white scale bars are 100 μm.

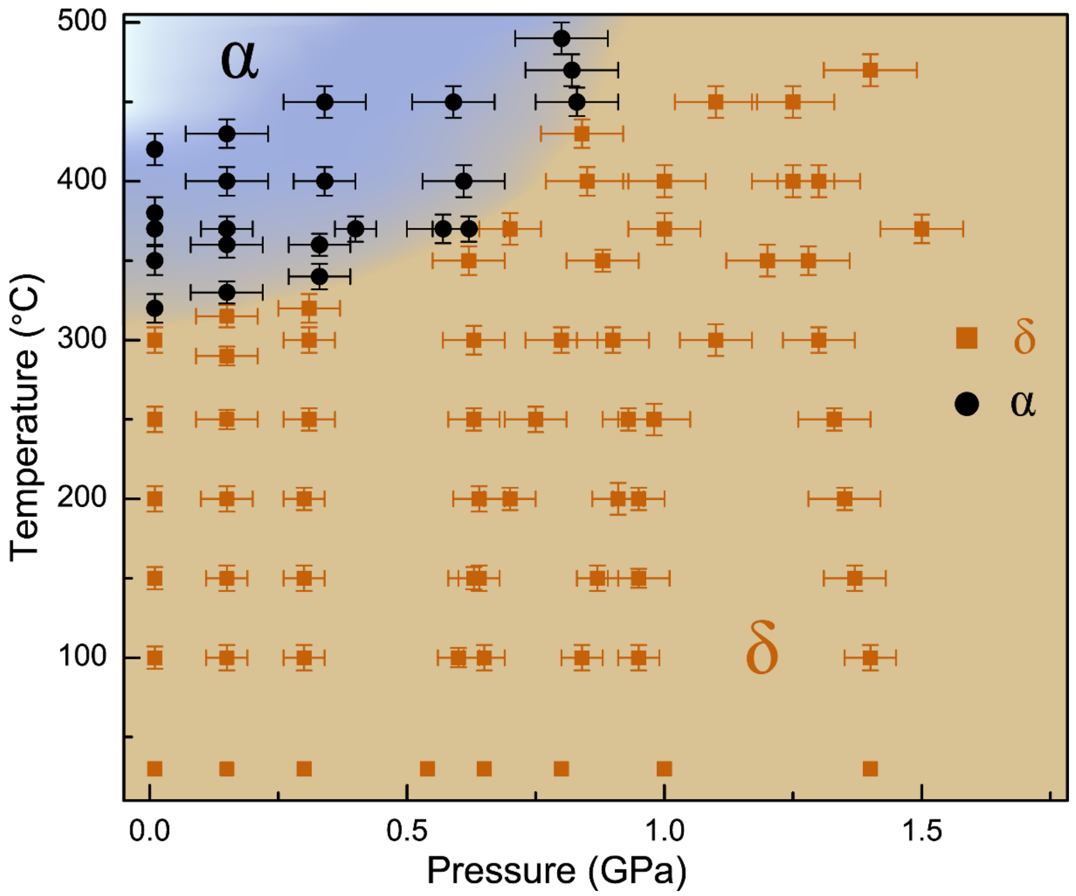

Supplementary Figure 2. *P*-*T* phase diagram of $CsPbI_3$. The yellow and blue background represent the δ and α phase, respectively. XRD measurements indicated that the sample starts to melt above 440 ºC at ambient pressure, and the melting temperature increases with pressure. For all of our *P*-*T* runs, $CsPbI_3$ is kept in a crystalline state and undergoes solid-to-solid phase transitions. The temperature error bars are determined based on the temperature fluctuation before and after measurements, which further results in the pressure uncertainty (see Methods for details).

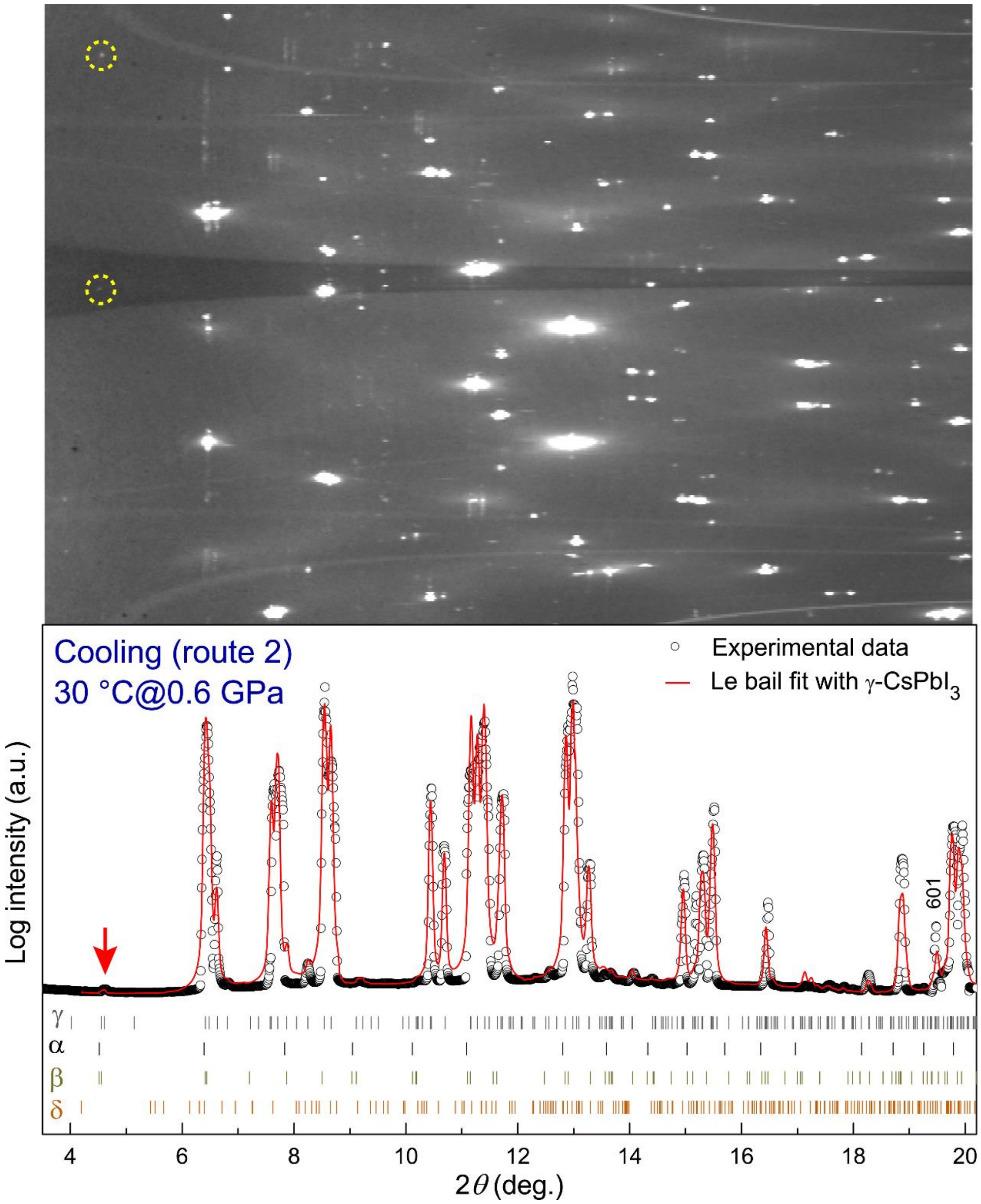

Supplementary Figure 3. Caked and integrated XRD pattern (λ = 0.4959 Å) of the preserved black $CsPbI_3$ phase in Fig. 2b shown on a log scale. The open circles are the experimental data and the red solid line is the fitted curve using the Le Bail method in the GSAS software package[1] with the γ-$CsPbI_3$ structure. The vertical tick marks indicate the peak positions of the γ, α, β, and δ phases. The yellow dashed circles and the red arrow indicate a weak peak at a low diffraction angle.

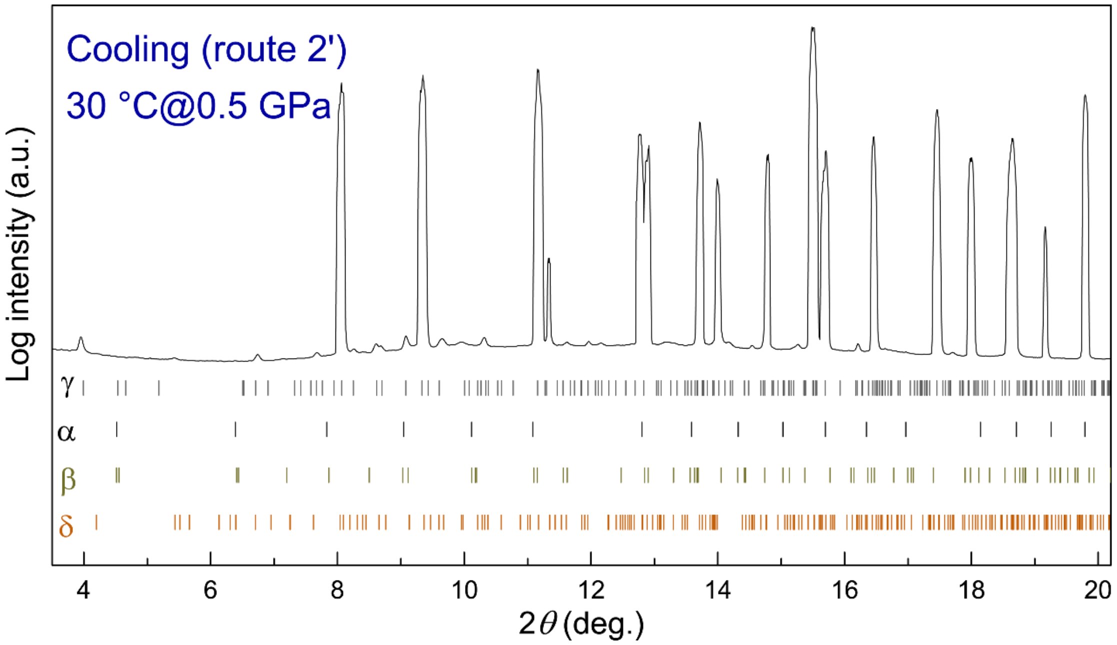

Supplementary Figure 4. XRD pattern (λ = 0.4959 Å) of the preserved black $CsPbI_3$ phase in Fig. 2c shown on a log scale.

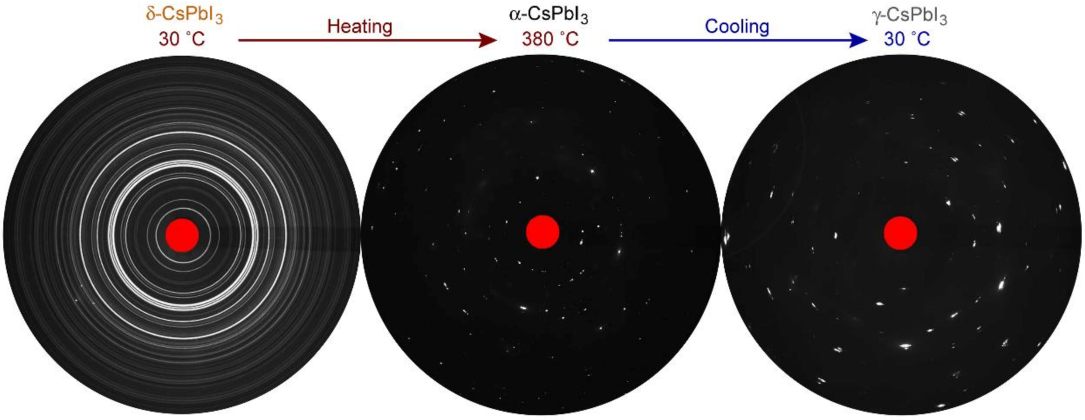

Supplementary Figure 5. The evolution of the two-dimensional diffraction images of $CsPbI_3$ at 0.6 GPa along heating and cooling cycle. The left, middle, and right images are δ-$CsPbI_3$ at 30 ˚C, α-$CsPbI_3$ at 380 ˚C, and γ-$CsPbI_3$ at 30 ˚C, respectively.

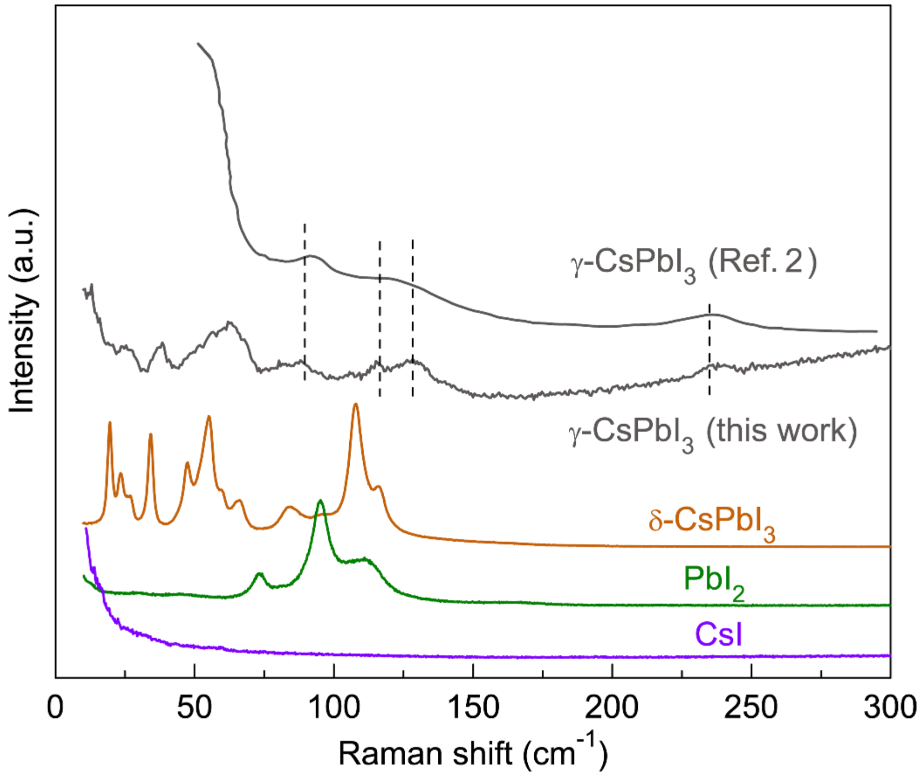

Supplementary Figure 6. Comparison of the Raman spectra of the preserved γ-$CsPbI_3$ phase with the precursors and the starting δ-$CsPbI_3$ phase at room temperature and ambient pressure. The Raman spectrum of the preserved black phase is similar to that of γ-$CsPbI_3$ synthesized by a solid-state method[2]. No Raman modes or XRD peaks from CsI or PbI_2 precursors are observed, indicating no decomposition of $CsPbI_3$.

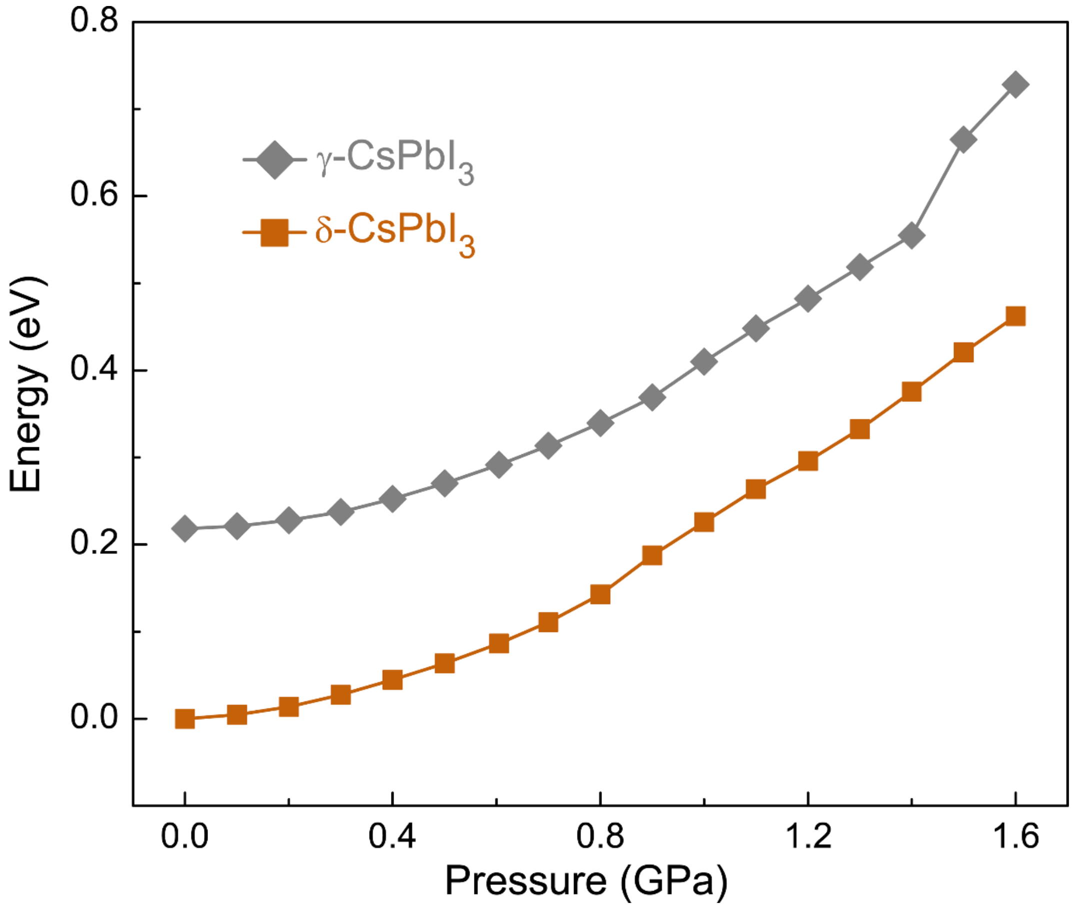

Supplementary Figure 7. Simulated total energy difference per unit cell (relative to the total energy of δ-$CsPbI_3$ at 0 GPa) of γ- and δ-$CsPbI_3$ as a function of pressure.

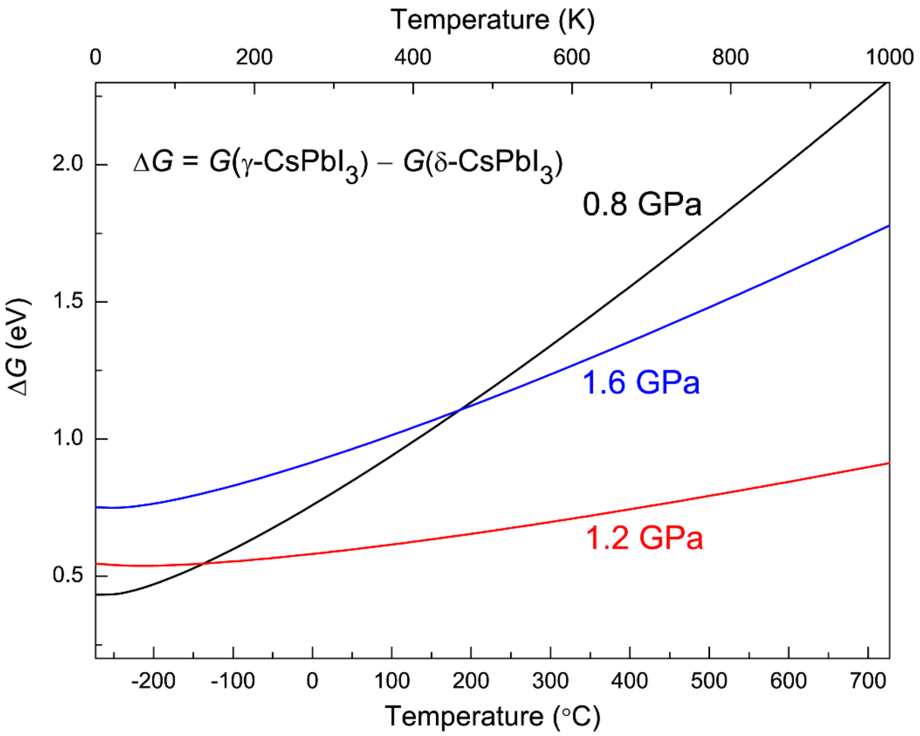

Supplementary Figure 8. The Gibbs free energy difference per unit cell between γ- and δ-$CsPbI_3$ ($\Delta G = G_\gamma - G_\delta$) as a function of temperature at 0.8, 1.2 and 1.6 GPa.

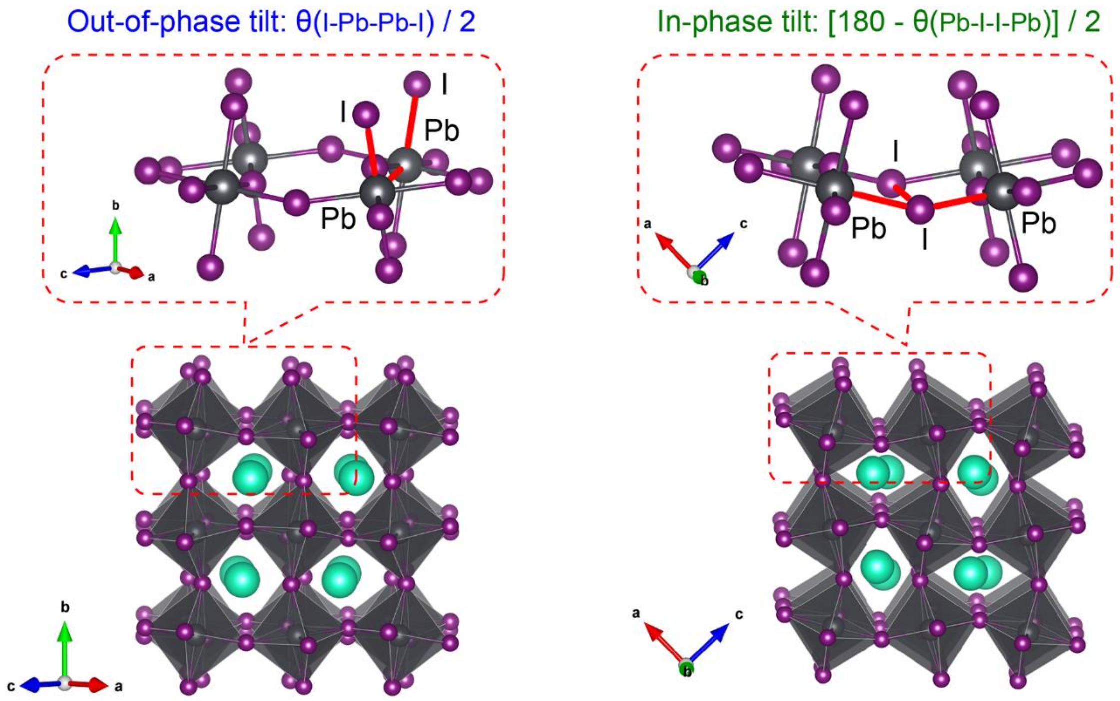

Supplementary Figure 9. The out-of-phase tilt and the in-phase tilt defined in our study based on the Glazer notation[3,4]. θ represents the torsion angle.

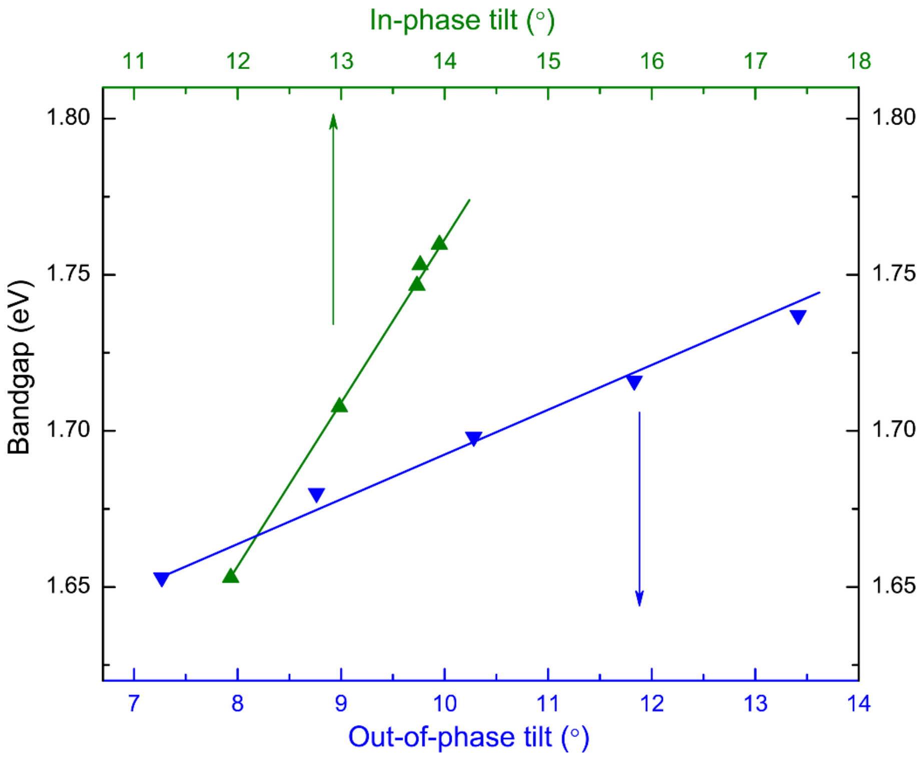

Supplementary Figure 10. The bandgap evolution as a function of the in-phase and out-of-phase tilt.

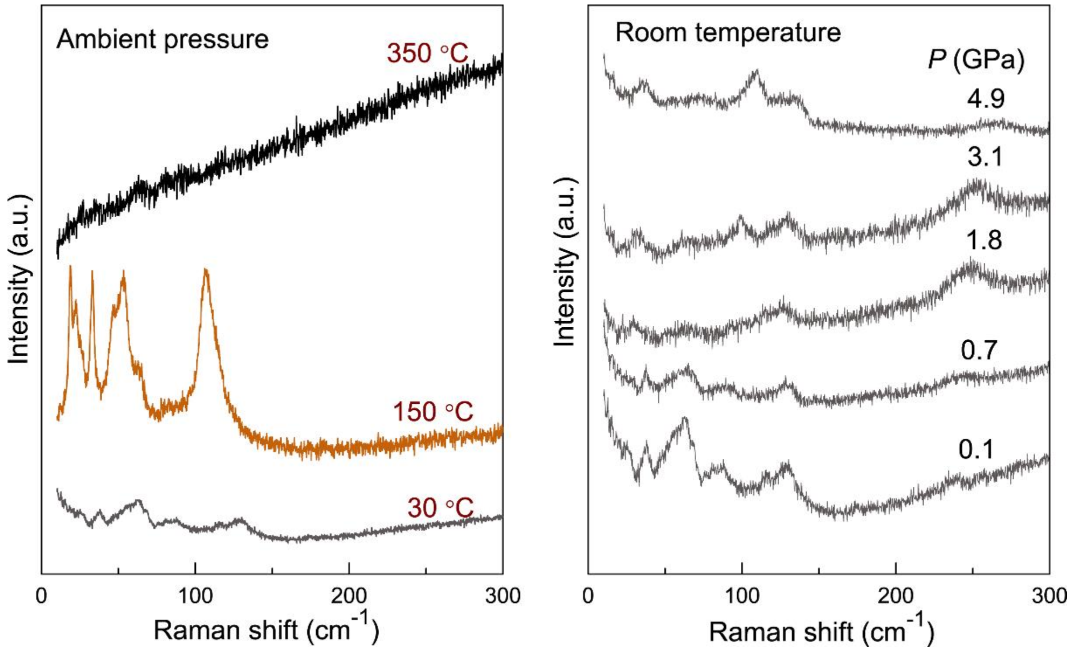

Supplementary Figure 11. Stability tests for the preserved γ-$CsPbI_3$ at high-temperature (left) and high-pressure conditions (right). The preserved γ-$CsPbI_3$ starts to convert to the δ-$CsPbI_3$ phase above 100 ˚C and further to the α-$CsPbI_3$ phase above 320 ˚C. With application of pressure at room temperature, the Raman spectra of γ-$CsPbI_3$ remain unchanged except for the blue shift of the Raman modes and changes in the relative intensity up to 4.9 GPa, above which the Raman intensity decreases and becomes indistinguishable.

Supplementary Table 1. The calculated *TS* of γ- and δ-$CsPbI_3$ from 300 to 570 K at 0.8, 1.2 and 1.6 GPa.

	0.8 GPa		1.2 GPa		1.6 GPa	
T (K)	TS_γ (eV)	TS_δ (eV)	TS_γ (eV)	TS_δ (eV)	TS_γ (eV)	TS_δ (eV)
300	3.08762	3.46065	3.22829	3.27326	3.02295	3.21353
310	3.23424	3.62503	3.38178	3.43007	3.16801	3.36825
320	3.38227	3.791	3.53675	3.58842	3.3145	3.5245
330	3.53166	3.95849	3.69315	3.74827	3.46238	3.68224
340	3.68238	4.12746	3.85094	3.90955	3.6116	3.84142
350	3.83437	4.29787	4.01008	4.07224	3.76212	4.00201
360	3.98762	4.46968	4.17052	4.23629	3.91391	4.16395
370	4.14207	4.64285	4.33224	4.40166	4.06693	4.32722
380	4.29771	4.81734	4.4952	4.56832	4.22115	4.49177
390	4.45449	4.99311	4.65936	4.73623	4.37653	4.65757
400	4.61238	5.17013	4.82469	4.90536	4.53304	4.82459
410	4.77137	5.34837	4.99116	5.07568	4.69066	4.99279
420	4.93141	5.52781	5.15876	5.24716	4.84936	5.16216
430	5.0925	5.70841	5.32744	5.41977	5.00912	5.33265
440	5.25459	5.89014	5.49718	5.59349	5.1699	5.50425
450	5.41768	6.07298	5.66796	5.76828	5.33168	5.67693
460	5.58173	6.2569	5.83975	5.94413	5.49444	5.85066
470	5.74673	6.44188	6.01254	6.12102	5.65817	6.02543
480	5.91265	6.6279	6.1863	6.29891	5.82284	6.2012
490	6.07948	6.81494	6.36102	6.4778	5.98842	6.37796
500	6.24719	7.00297	6.53666	6.65765	6.15491	6.55569
510	6.41578	7.19198	6.71322	6.83846	6.32228	6.73437
520	6.58521	7.38194	6.89067	7.02019	6.49051	6.91398
530	6.75549	7.57284	7.069	7.20284	6.65959	7.09449
540	6.92658	7.76465	7.24819	7.38638	6.82951	7.27591
550	7.09848	7.95737	7.42822	7.5708	7.00024	7.4582
560	7.27117	8.15098	7.60909	7.75608	7.17178	7.64135
570	7.44463	8.34545	7.79077	7.94222	7.3441	7.82535

References

1 Toby, B. H. & Von Dreele, R. B. GSAS-II: the genesis of a modern open-source all purpose crystallography software package. *J. Appl. Crystallogr.* **46**, 544-549 (2013).

2 Straus, D. B., Guo, S. & Cava, R. J. Kinetically stable single crystals of perovskite-phase $CsPbI_3$. *J. Am. Chem. Soc.* **141**, 11435-11439 (2019).

3 Glazer, A. M. The classification of tilted octahedra in perovskites. *Acta. Cryst.* **B28**, 3384-3392 (1972).

4 Woodward, P. M. Octahedral tilting in perovskites. II. Structure stabilizing forces. *Acta. Cryst.* **B53**, 44-46 (1997).
